# Integrated genomic and experimental assessment of the biosafety of *Paraburkholderia sacchari* as a promising microbial chassis

**DOI:** 10.1007/s11274-026-05227-y

**Published:** 2026-09-26

**Authors:** Matheus Marques Torres, João Henrique Diniz Gervasio, Eric André Velasco Yépez, Amanda Silva Santos, Gabriele Muniz Costa, Maria Luisa Zardo, Juliana Cardinali-Rezende, Welington Luiz de Araújo, José Gregório Cabrera Gomez, Luiziana Ferreira da Silva

**Affiliations:** 1 Laboratory of Bioproducts, Department of Microbiology, Institute of Biomedical Sciences, University of São Paulo, São Paulo, SP Brazil; 2 S2B - Synthetic and Systems Biology Center, INOVA, University of São Paulo, São Paulo, SP Brazil; 3 Model-Based Evolutionary Genomics Unit, Okinawa Institute of Science and Technology, Onna, Okinawa-ken Japan; 4 LABMEM - Department of Microbiology, Institute of Biomedical Sciences, University of São Paulo, São Paulo, SP Brazil; 5 LGEM - Laboratory of Genetics and Metabolic Engineering, Center for Natural and Human Science, Federal University of ABC, Santo André, SP Brazil

**Keywords:** *Burkholderia sacchari*, Genome sequencing, Microbial biosafety, Microbial chassis, *Paraburkholderia sacchari*

## Abstract

*Paraburkholderia sacchari* is an environmental member of the Burkholderiaceae with recognized biotechnological potential, yet its genomic diversity, biosafety profile, and evolutionary relationship to pathogenic *Burkholderia* species remain poorly understood. Here, we present a comprehensive genomic and phenotypic characterization of *P. sacchari* strain LMG 19450^T^ and compare it with three additional *P. sacchari* genomes and phylogenetically related *Burkholderia* species. A high-quality genome assembly confirmed its placement within the *Paraburkholderia* clade and species-level relatedness among *P. sacchari* strains despite detectable intraspecies genomic variability. Pangenome analysis revealed a large, conserved core genome and an extensive accessory gene repertoire, consistent with adaptation to plant-associated soil environments. Comparative analysis of virulence-associated genes showed a marked contrast between *P. sacchari* and pathogenic *Burkholderia*, with the former lacking most canonical pathogenicity determinants. Gene tree–species tree reconciliation further indicated a constrained horizontal gene transfer profile for virulence-associated gene families, while genes involved in transcriptional regulation displayed higher transfer propensity. Antimicrobial resistance profiling revealed a limited resistome dominated by efflux-associated homologs, consistent with broad phenotypic susceptibility. Notably, strain LMG 19450^T^ exhibited antibiotic susceptibility patterns comparable to those of the established microbial chassis *Pseudomonas putida* KT2440. Virulence assessment using the *Galleria mellonella* model demonstrated markedly attenuated pathogenicity relative to *Burkholderia cepacia*. Together, these findings support *P. sacchari* as an environmentally adapted bacterium with a favorable biosafety profile and highlight the value of integrating genomic and experimental approaches to evaluate emerging microbial chassis.

**Supplementary Information:**

The online version contains supplementary material available at https://doi.org/10.1007/s11274-026-05227-y.

## Introduction

The development of microbial platforms as chassis for the sustainable and efficient production of industrially relevant molecules has become a central focus in modern biotechnology. Microbial chassis provide standardized and engineerable biological frameworks capable of hosting complex biosynthetic pathways for production of bioplastics, biofuels, pharmaceuticals, fine chemicals, and other high-value compounds (Nielsen and Keasling 2016; Chi et al. 2019; Xu et al. 2020). Beyond productivity, an effective microbial chassis must combine genetic tractability, metabolic versatility, robustness under controlled cultivation conditions, and long-term stability during industrial operation (Lee et al. 2019; Chubukov et al. 2016).

Among these requirements, biosafety has emerged as a decisive criterion for chassis selection, particularly as engineered microorganisms move beyond contained laboratory environments into pilot-scale facilities, industrial plants, and, in some cases, open or semi-open systems. The use of organisms classified as biosafety level 1 (BSL-1) reduces regulatory complexity, facilitates licensing procedures, and minimizes infrastructure costs associated with containment and operator protection (WHO - World Health Organization 2020; National Institute of Health 2024). Consequently, there is a growing interest in identifying environmental bacteria that combine favorable metabolic traits with demonstrably low pathogenic potential, thereby expanding the repertoire of safe alternatives beyond traditional model organisms such as *Escherichia coli* and *Saccharomyces cerevisiae* (Lorenzo et al. 2018; Calero and Nikel 2018).

In this context, *Paraburkholderia sacchari* (formerly classified as *Burkholderia sacchari*) has attracted attention as a promising candidate for chassis development. Originally isolated from sugarcane soil, *P. sacchari* is a Gram-negative, aerobic bacterium characterized by an unusually high carbon flux toward polyhydroxyalkanoate (PHA) synthesis, enabling efficient bioplastic production from renewable substrates (Gomez et al. 1996; Brämer et al. 2001; Silva et al. 2004; Oliveira-Filho et al. 2022). The species exhibits notable metabolic plasticity, including the utilization of diverse carbon sources such as sugars, organic acids, and agro-industrial residues, making it particularly attractive for circular bioeconomy applications (de Andrade et al. 2017; Cesário et al. 2014; Silva et al. 2004, 2020). However, despite its biotechnological relevance, the biosafety status of *P. sacchari* remains poorly characterized. Previous genotypic studies have reported broad antibiotic susceptibility profiles in *P. sacchari*, consistent with expectations for environmental soil bacteria rather than opportunistic pathogens (Alexandrino et al. 2015).

The broader taxonomic context of *P. sacchari* also necessitates careful evaluation. The genus *Burkholderia* historically encompassed a heterogeneous assemblage of species spanning plant-associated mutualists, environmental saprophytes, and clinically significant opportunistic pathogens, including members of the *Burkholderia cepacia* complex (Bcc) (Mahenthiralingam and Vandamme 2005; Eberl and Vandamme 2016). This taxonomic breadth has complicated risk assessment, as environmental isolates may be perceived as unsafe due to their association with pathogenic relatives. Phylogenomic analyses over the past decade have resolved much of this ambiguity, demonstrating that pathogenic and environmental species form deeply separated evolutionary lineages. These insights led to the proposal of the genus *Paraburkholderia*, which groups predominantly non-pathogenic, plant-associated species formerly classified within *Burkholderia* (Sawana et al. 2014; Dobritsa and Samadpour 2016; Estrada-de los Santos et al. 2018). Comparative genomic studies consistently place *P. sacchari* close to the *Paraburkholderia* clade, highlighting the absence of key virulence operons and pathogenicity islands characteristic of clinical *Burkholderia sensu stricto* isolates.

Establishing the biosafety of candidate chassis organisms within such taxonomically complex groups requires a comprehensive, genome-informed approach. Advances in whole-genome sequencing and comparative genomics have enabled systematic screening for genes associated with virulence, antibiotic resistance, secretion systems, toxins, and mobile genetic elements that may increase pathogenic potential (Su et al. 2018; Holý et al. 2023; Zhang et al. 2025). Resources such as the Virulence Factor Database (VFDB) and the Comprehensive Antibiotic Resistance Database (CARD) have become indispensable for contextualizing genomic content within known pathogenic frameworks. However, in silico predictions alone are insufficient, as gene presence does not necessarily translate into phenotypic behavior. Accordingly, biosafety assessments increasingly integrate genomic analyses with experimental validation, including antimicrobial susceptibility testing and infection models that provide functional insight into host–microbe interactions (Desbois and Coote 2011; Tsai et al. 2016).

In this study, we present a comprehensive biosafety evaluation of *Paraburkholderia sacchari* LMG 19450^T^ based on a newly generated, high-quality genome assembly. This assembly achieves 99.98% completeness and resolves genomic regions that were previously fragmented or ambiguous, enabling precise assessment of virulence-associated loci, resistance determinants, and mobile genetic elements. By integrating comparative genomics, pangenome analysis, horizontal gene transfer profiling, antimicrobial susceptibility testing, and in vivo virulence assays, we aim to establish a robust and multidimensional biosafety profile for *P. sacchari*. Through this approach, we provide a framework for evaluating environmental bacteria as microbial chassis and contribute to the rational expansion of safe and metabolically versatile platforms for industrial biotechnology.

## Materials and methods

### Bacterial strain and growth conditions

The bacterial strain used in this study was originally isolated in Brazil through a bioprospecting program carried out in soil from a sugarcane plantation (Brämer et al. 2001). This strain was later identified as *Burkholderia sacchari* (Brämer et al. 2001) and more recently *Paraburkholderia sacchari *(DSM 17165) and was used as the test strain. The control strains were obtained from the American Type Culture Collection (ATCC) and identified as *Burkholderia cepacia* B008/25,416 (ATCC 25416) and *Pseudomonas putida* KT2440 (ATCC 47054). Both *Burkholderia* and *Pseudomonas* strains were cultured in lysogeny broth (LB) medium and incubated at 37 °C for 24 h. Subsequently, the cultures were centrifuged at 4000 rpm for 10 min and resuspended in phosphate-buffered saline (PBS) to an optical density at 600 nm (OD₆₀₀) of 1.0. Such suspensions were further used for the antibiogram assays and for stock maintenance on solid LB medium at 4 °C.

### Sequencing and genome assembly

For the sequencing of *P. sacchari* LMG 19450ᵀ genome, total genomic DNA was extracted using the Wizard Genomic DNA Purification Kit (Promega), quantified with a Qubit fluorometer (Qubit 4 Fluorometer), and assessed for integrity by 0.8% agarose gel electrophoresis. The sequencing library was prepared using the Rapid Barcoding Kit (Oxford Nanopore Technologies). Sequencing was performed on a MinION Mk1C device running MinKNOW software (v23.07.9) for 72 h. Raw FAST5 files were converted to the POD5 format using the POD5 tools (Oxford Nanopore Technologies 2024a). Basecalling was subsequently performed with Dorado (v2.1.0; Oxford Nanopore Technologies 2024b) using the super-accuracy (sup) model, generating FASTQ files for downstream analyses. The long reads were assembled de novo using Flye v2.9.6 (Kolmogorov et al. 2019) with the --nano-hq option and an estimated genome size of 7.2 Mb. Previously generated Illumina short reads from the same strain (Alexandrino et al. 2015) were aligned to the Nanopore assembly using BWA-MEM v0.7.17 (Li 2013), and the resulting alignments were used to polish the assembly with Polypolish v0.6.0 (Wick and Holt 2022). The revised genome assembly generated in this study has been deposited in GenBank as an updated version of the previously available assembly (GCA_000785435.2) and is associated with BioSample accession number SAMN03120802. This represents the most recent genome assembly generated for this strain in 2026. 

### Phylogenetic analysis and reconciliation

To reconstruct the phylogenetic tree of the genera *Burkholderia*, we opted to go the classical route using rRNA 16 S as a marker gene. The genomic data for all 286 complete genomes from The *Burkholderia* Genome Database (Winsor et al. 2008) was downloaded on 26 March 2025 (Supplementary material – Table S1). Gene prediction for all downloaded genomes and the new assembly was done using prodigal (Hyatt et al. 2010), and 16 S rRNA gene search with barrnap (Tseemann, 2013). Different species may contain more than one copy of such genes, so to avoid paralogy when there was more than one identical copy, the parsimonious choice was made, and the most common copy was selected. To root the *Burkholderia* tree, two bacterial genera were used as outgroups, *Pseudomonas* and *Ralstonia*. For each group, eleven (11) 16 S rRNA gene sequences were obtained from RNACentral (Sweeney et al. 2018). Ribosomal RNA 16 S sequences from the group *Paraburkholderia* were also obtained from RNACentral, as the present genome is from a strain believed to be closer to *Paraburkholderia* than to *Burkholderia*. To resolve the phylogenetic tree a multiple sequence alignment of the 16 S rRNAs was performed using Clustal Omega (Sievers et al. 2014). The phylogenetic tree was created using IQ-TREE 2 (Minh et al. 2020) with JC+C60 + F substitution models, with 10,000 ultrafast bootstraps. For ease on visualization, we prune the final inferred tree showing just the members of *Burkholderia* and *Paraburkholderia*.

To have better confidence in the taxonomy indication of or sample, beyond the species three inferred from 16 S RNA sequences, we inferred a tree with 110 markers and pruned the bacteria species tree from Genome Taxonomy Database (GTDB) (Parks et al. 2021). We pruned the GTDB tree in the level of family Burkholderiaceae and chose the deepest branch where *Burkholderia* and *Paraburkholderia* differentiate. The markers used to infer the tree were chosen based on the GTDB markers that we could find in the 330 genomes we analyzed. The NCBI accession code and the list of markers is in the supplementary material (Tables S4 and S6). We performed a multiple sequence alignment (msa) using ClustalO (Sievers and Higgins 2017), with default parameters, for each marker gene family. We proceed by concatenating those msa for every genome and used that resulting fasta file as input for iqtree3 (Kalyaanamoorthy et al. 2017). IQtree3 tested 968 DNA mixture models and proceeded the inference with the best fit model (GTR + F+R10) using the ultrafast bootstrap (Hoang at al. 2017) with 10,000 trees. For ease on visualization, we prune the final inferred tree showing just the members of *Burkholderia*, *Paraburkholderia*, and *Trinickia*.

A group of 41 genes were selected to verify how their evolution happened in the *Burkholderia* group, that is, reconciliation events of origination, speciation, duplication, loss and horizontal gene transfer to the species tree. The ortholog amino acid sequences were obtained by searching for their name at the Burkholderia Genome Database. For each gene family, 10.000 phylogenetic gene trees were constructed as a result of IQ-TREE ultrafast bootstrap with LG+C60 + F substitution model. AleRax (Morel et al. 2024) was used to calculate the reconciliation.

### Genomic analysis

Genomic analyses were performed using the updated genome assembly of Paraburkholderia sacchari LMG 19450ᵀ generated from long-read sequencing followed by polishing with previously generated Illumina short reads. Genomic annotation was performed using the RAST server (Aziz et al. 2008). The annotated genome was used for operon identification, metabolic pathway reconstruction, and comparative genomic analyses. To detect genomic islands associated with virulence and lateral gene transfer, the genome was scanned using the Pathogenicity Island Database (PAIDB v2.0) (Yoon et al. 2014), and the IslandViewer 4 platform (Bertelli et al. 2017) using the default parameters of each platform unless otherwise specified. These tools integrate sequence composition-based and comparative genomics approaches to identify regions likely acquired by horizontal gene transfer and enriched in virulence-associated genes. Genes associated with antimicrobial resistance were identified using the Comprehensive Antibiotic Resistance Database (CARD v3.2.4) (Jia et al. 2017) and ResFinder (v4.7.2) (Zankari et al. 2012). Candidate resistance genes were considered only when sequence identity and coverage exceeded the confidence thresholds recommended by each platform. Virulence-associated genes were identified using the Virulence Factor Database (VFDB, v6.0) and further evaluated using the PATRIC database (v3.2.76). Genes involved in classical virulence mechanisms, including type III and type VI secretion systems, toxins, and adhesins, were examined within their genomic context and operon organization to assess their conservation and potential acquisition by horizontal gene transfer. Comparative genomic analyses between *P. sacchari* and phylogenetically related species within the genera *Burkholderia* and *Paraburkholderia* were performed to identify conserved and variable virulence-associated loci, contributing to the taxonomic characterization and biosafety assessment of the strain. Pan-genome analysis was performed locally using Anvi’o v8 (Eren et al. 2021) with the anvi-pan-genome workflow and default parameters unless otherwise specified. Average Nucleotide Identity (ANI) and digital DNA–DNA hybridization (dDDH) values were calculated using the Type (Strain) Genome Server (TYGS) (Meier-Kolthoff and Göker 2019) with the default parameters implemented by the platform. Heatmaps and graphical representations of pathogenicity-associated genes were generated using R v4.4.3 with the ComplexHeatmap (v2.20.0) (Gu et al. 2016) and ggplot2 (v4.0.3) (Wickham 2016) packages.

### *Galleria mellonella* infection model assay and statistical analysis

For the assessment of pathogenicity, *Galleria mellonella* larvae were reared under laboratory conditions as previously described (Gonçalves et al. 2019) and maintained on a diet consisting of honeycombs and an artificial food mixture composed of wheat flour, corn flour, dry yeast, milk powder, glycerol and honey. Larvae were maintained in sterile 150-mm Petri dishes at 28 °C until reaching the fourth instar stage. For inoculation, larvae were randomly assigned to experimental groups of ten individuals and maintained in new sterile 150-mm Petri dishes according to their respective treatment groups.

For each experimental group, three independent replicates of ten larvae each were used. Each larvae group was infected with one of the bacterial strains tested (*Burkholderia cepacia*, *Pseudomonas putida*, or *Paraburkholderia sacchari*). Bacterial cells were harvested, washed, and resuspended in sterile phosphate-buffered saline (PBS) prior to inoculation. Bacterial suspensions were adjusted to 10⁶ or 10⁸ CFU/mL, and 10 µL were injected into each larva, corresponding to inocula of approximately 10⁴ and 10⁶ CFU per larva, respectively. Inoculations were performed by injecting 10 µL of the bacterial suspension into the second-to-last proleg on the left side of each larva using a 10 µL Hamilton syringe fitted with a 26-gauge needle. Control groups consisted of larvae injected with 10 µL of sterile PBS, as well as a non-injected group to control for handling-related mortality. After injection, larvae were incubated at 28 °C in the dark and monitored every 12 h for survival over a period of 144 h. Larvae were considered dead when they failed to respond to gentle physical stimulation. The entire experiment was independently repeated on three separate occasions. Statistical analyses were performed using R v4.5.3. Kaplan–Meier survival curves were generated using the survminer (v0.5.2) package, and pairwise comparisons between survival curves were performed using log-rank tests with Benjamini–Hochberg correction for multiple comparisons. Data manipulation was conducted using the dplyr (v1.2.1) and tidyr (v1.3.2) packages, and graphical representations were produced using ggplot2 (v4.0.3). Statistical significance was considered at *P* < 0.05.

### Antibiotic susceptibility testing of *P. sacchari*

Antimicrobial susceptibility testing was performed according to the CLSI methodology (Hudzicki 2009) using the Kirby–Bauer disk diffusion method. For susceptibility analysis, commercial antibiotic disks (CECON Control Center and Diagnostic Products, SP, Brasil) were used at concentrations established by the EUCAST protocol for *Burkholderia pseudomallei* and by the CLSI protocol for the *Burkholderia cepacia* complex, including antibiotics commonly recommended for this complex, such as ceftazidime (CAZ, 30 µg), meropenem (MER, 10 µg), tetracycline (30 µg), levofloxacin (LVX, 5 µg), and trimethoprim–sulfamethoxazole (SUT/SXT, 1.25/23.75 µg).

In addition, amoxicillin–clavulanic acid (AMC, 20 µg), ciprofloxacin (CIP), imipenem (IPM, 10 µg), nalidixic acid (NAL), doxycycline (DOX, 30 µg), and penicillin (PEN) were also tested to demonstrate how this *Burkholderia* spp. (*Paraburkholderia spp*) species exhibits a distinct susceptibility/resistance profile within the complex. *Burkholderia cepacia* (B008/25416) was used as the control strain. Inhibition zone diameters were interpreted according to the Clinical and Laboratory Standards Institute (CLSI) standards Breakpoint Tables.

For the antimicrobial susceptibility test, the strains were initially cultured on Muller-Hinton agar medium (MHA, Himedia) and then transferred to Muller-Hinton broth and cultivated for 18 h at 30 °C. After growth, an OD_600_ of 0.1 (compatible with the N^o^. 0.5 in McFarland standard) was used, and 100 µL was spread over the MHA medium using a Drigalski loop. Antibiotic discs were then added in triplicate and incubated at 30 °C for 18 h. After this period, the presence of the inhibition halo was analyzed to understand the resistance pattern of *B. sacchari.*

### Determination of the minimum inhibitory concentration of antibiotics on *P. sacchari*

Minimum inhibitory concentrations (MICs) were determined by the broth microdilution test using sterile 96-well microplates following CLSI-based methodology adapted for environmental Gram-negative bacteria. The assays were performed using *Paraburkholderia sacchari* LMG 19450^T^, *Burkholderia cepacia* B008/25,416, and *Pseudomonas putida* KT2440. Bacterial strains were initially cultivated overnight on LB agar plates at 30 °C. Subsequently, five isolated colonies from each culture were transferred to Mueller–Hinton broth and cultivated until reaching an optical density at 600 nm (OD_600_) of approximately 0.4. The cultures were then adjusted to a final concentration of approximately 5 × 10^5^CFU mL^− 1^ for inoculation. The antibiotics were serial two-fold diluted on Mueller-Hinton broth in the ranges of piperacillin (128-0,125 µg/ml), meropenem (256-0,25 µg/ml), gentamicin (256-0,125 µg/ml), ciprofloxacin (16 − 0,125 µg/ml), doxycycline (4 − 0,125 µg/ml), chloramphenicol (64 –1 µg/ml) and fosfomycin (1024–16 µg/ml). Antibiotics were selected to represent clinically relevant classes and to complement the disk diffusion panel, with emphasis on agents commonly used as reference compounds in susceptibility surveillance of Gram-negative environmental bacteria. Plates were incubated at 30 °C for 18–24 h under orbital shaking (800 cpm) in a LogPhase 600 Microbiology Reader (BioTek Instruments, Winooski, VT, USA). MIC values were defined as the lowest antibiotic concentration showing complete absence of visible bacterial growth. All assays were performed in biological triplicate.

## Results

### Genome assembly and general genomic features of *Paraburkholderia sacchari*

The genome of *Paraburkholderia sacchari* LMG 19450^T^, isolated from sugarcane soil, was assembled into five contigs, with a total length of 7,377,658 bp and a GC content of 63.98%. A total of 6,577 protein-coding sequences (CDSs) were predicted, together with 71 tRNA genes and 14 rRNA genes. Assembly contiguity was high, with an N50 value of 2.1 Mb. Genome completeness, assessed using CheckM, reached 99.98%, indicating a near-complete genome. To place strain LMG 19450^T^ in the context of other available *P. sacchari* genomes, its genomic features were compared with three additional *P. sacchari* strains recently deposited in public databases, isolated from different plant-associated soils (Table 1).

**Table 1:** General genomic characteristics of *Paraburkholderia sacchari* LMG 19450^T^ and three other soil-isolated *P. sacchari* strains, whose genomes are deposited in public databases

Strain	Genome size (bp)	GC (%)	Contigs	N50	CDS	tRNA	rRNA	Completeness (%)	Isolation source	Reference
*P. sacchari* LMG 19450^T^	7,377,658	63.98	5	2.1 Mb	6,577	71	14	99.98	Sugarcane soil	Gomez et al. 1996
*P. sacchari* PAR1	8,053,473	64.07	5	2.4 Mb	7,065	71	14	99.10	Tomato soil	Li et al. 2024 BioProject - PRJNA1115909
*P. sacchari* Suichang 626	8,100,940	64.07	5	2.2 Mb	7,167	76	14	99.10	Bamboo root soil	Wang et al. 2021
*P. sacchari* ABIP2689	7,647,997	64.05	45	393.3 kb	6,762	61	3	99.41	Rice soil	Wallner et al. 2021

The four *P. sacchari* genomes exhibited similar genome sizes (7.65–8.10 Mb), GC contents (~ 64%), and high completeness (> 99%), supporting their suitability for comparative genomic analyses. The main differences were observed in assembly contiguity, with strains PAR1 and Suichang 626 assembled into five contigs, whereas strain ABIP2689 remained more fragmented (45 contigs). Additional assembly and annotation statistics are summarized in Table 1.

### Phylogenetic placement and genome-wide relatedness of *Paraburkholderia sacchari*

**Fig. 1:**
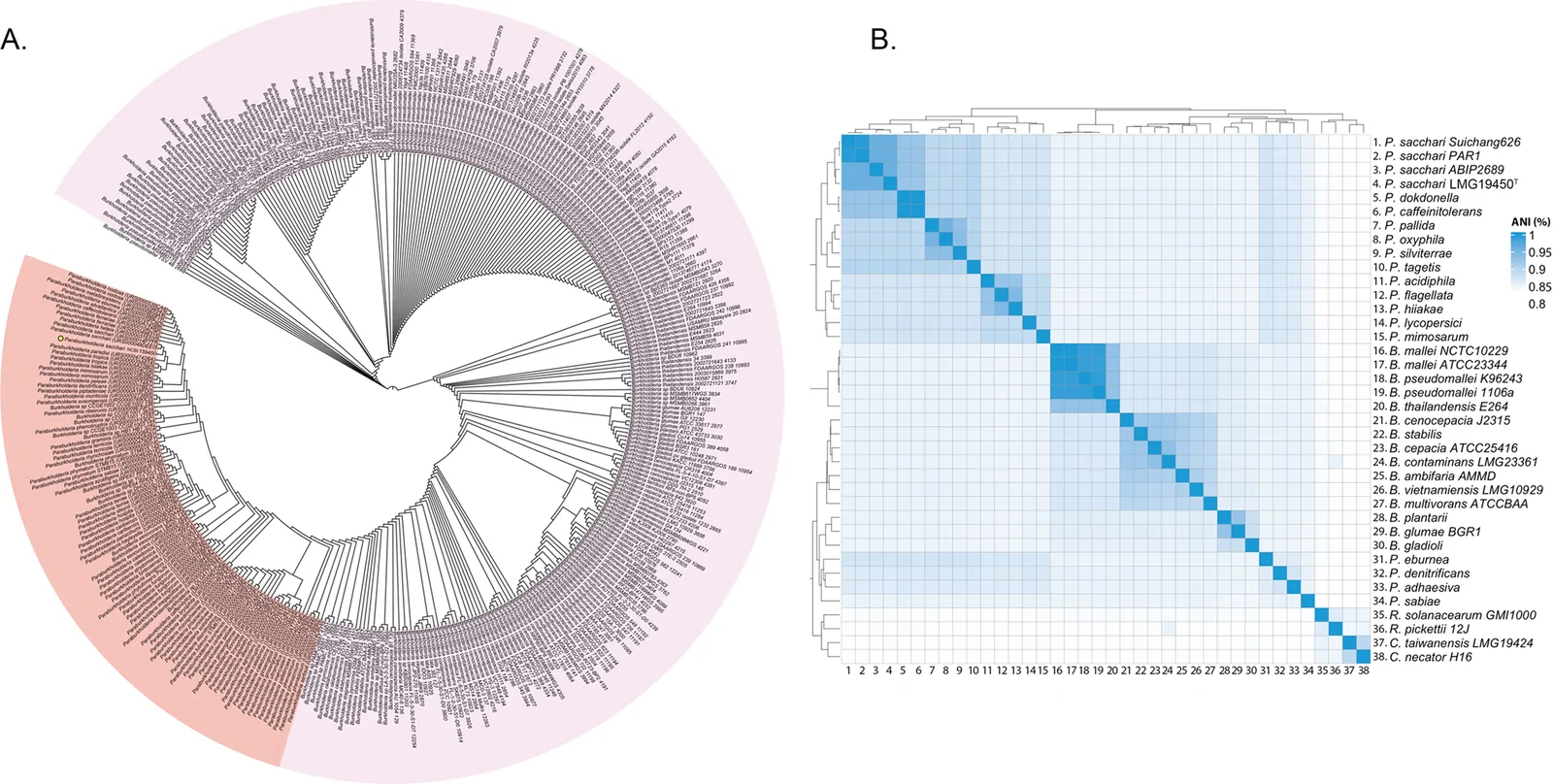
(**A**) Maximum-likelihood phylogenetic tree inferred from 16 S rRNA gene sequences of representative species from the genera *Burkholderia* and *Paraburkholderia*. Colored sectors indicate the major clades, with the Burkholderia clade shown in pink and the Paraburkholderia clade shown in orange. *Paraburkholderia sacchari* LMG 19450ᵀ strain investigated in this study is highlighted by a yellow circle. (**B**) ANI heatmap based on ANIm values, including hierarchical clustering. Abbreviations: P., *Paraburkholderia*; B., *Burkholderia*; R., *Ralstonia*; C., *Cupriavidus*

The phylogenetic relationships within the genus *Burkholderia* were evaluated using a 16 S rRNA-based phylogeny that included 286 complete genomes from the *Burkholderia* Genome Database, complemented with representative sequences from *Paraburkholderia* species (Fig. 1A).

In this analysis, the four *P. sacchari* strains formed a coherent cluster that was clearly separated from pathogenic *Burkholderia* species and grouped within the environmental *Paraburkholderia* lineage. High branch support values were observed across major nodes, supporting the robustness of the inferred topology. Genome-wide phylogenetic reconstructions based on GTDB marker genes and core-genome sequences also produced highly consistent topologies, supporting the placement of *P. sacchari* within the genus *Paraburkholderia* and the relationships inferred from the 16 S rRNA gene analysis (Supplementary Figure S1).

Genome-wide relatedness among the four *P. sacchari* strains was further assessed using Average Nucleotide Identity (ANI) based on MUMmer alignments (ANIm). The ANI dataset included representative pathogenic *Burkholderia* species and *Paraburkholderia* species positioned close to *P. sacchari* in the 16 S rRNA phylogeny (Fig. 1A). Pairwise ANI values between strain LMG 19450^T^ and the other *P. sacchari* genomes were 96.30% for strain PAR1, 96.28% for strain Suichang626, and 96.27% for strain ABIP 2689 (Fig. 1B). Consistent clustering was observed in the hierarchical organization of the ANI heatmap, with the four *P. sacchari* genomes forming a distinct group separated from other *Burkholderia* and *Paraburkholderia* species included in the analysis.

Digital DNA–DNA hybridization (dDDH) values were calculated to further examine genomic relatedness at the species boundary (Supplementary data - Table S2). dDDH values between strain LMG 19450^T^ and strains Suichang626 and PAR1 were 70.0% and 70.8%, respectively, whereas the value between strain LMG 19450^T^ and ABIP2689 was 67.6%. Notably, comparisons among the non-reference strains revealed greater variability, with dDDH values of 88.9% between Suichang626 and PAR1, but markedly lower values between ABIP2689 and the other two strains (65.2% with Suichang626 and 64.9% with PAR1). While ANI consistently supported the classification of all four genomes as *P. sacchari*, the lower dDDH values observed for strain ABIP2689 place this strain near the lower boundary commonly used for species delineation.

### Pangenome overview and strain-specific gene content of *Paraburkholderia*

**Fig. 2:**
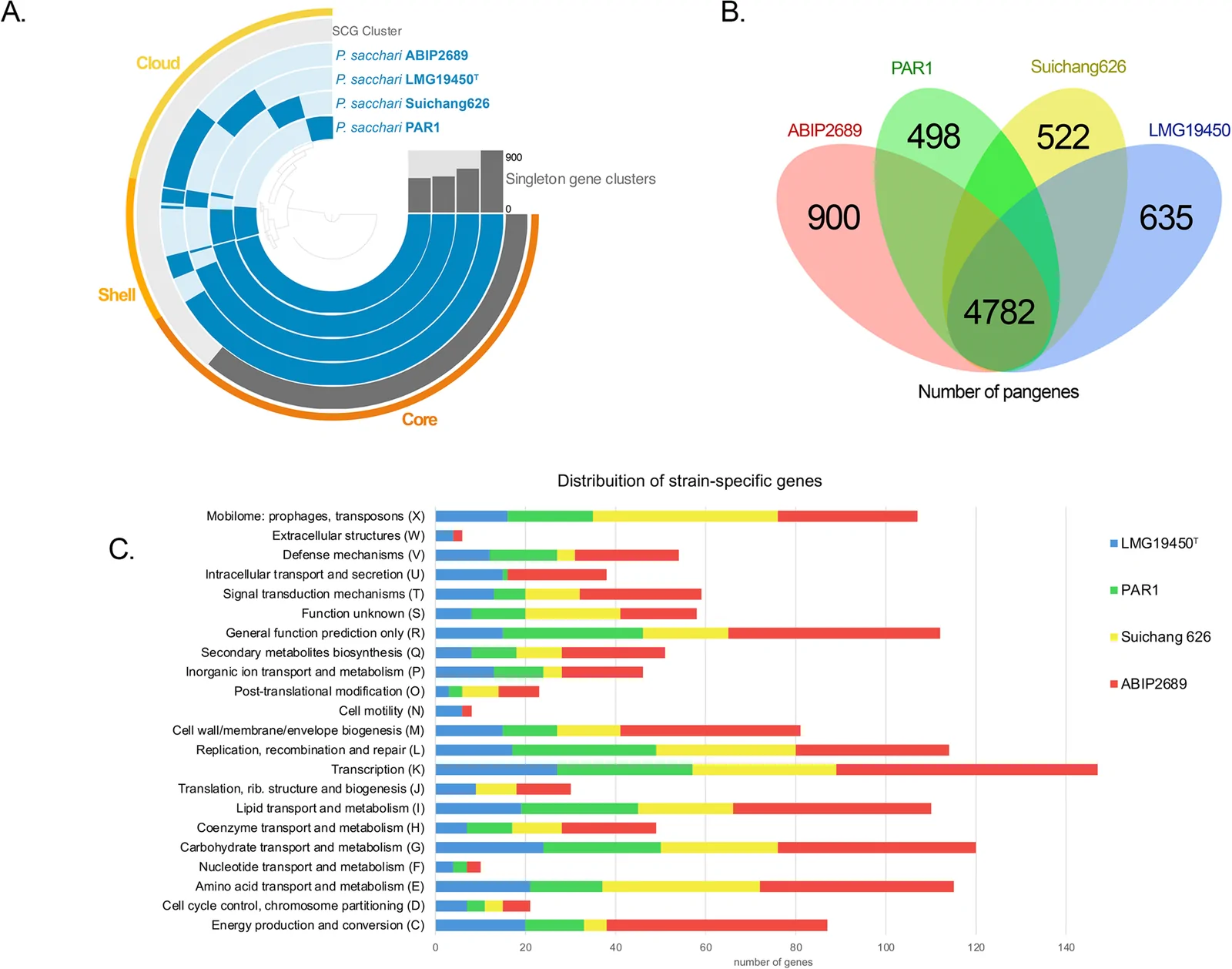
Pangenome architecture of *Paraburkholderia sacchari*. (**A**) anvi’o pangenome visualization across the four *P. sacchari* genomes. Genes were classified as core (present in all four genomes), shell (present in two or three genomes), or cloud (present in a single genome) according to Anvi’o pangenome classification. (**B**) Venn diagram illustrating the number of core and strain-specific gene clusters shared among the four genomes. (**C**) Horizontal bar chart showing the distribution of strain-specific (cloud) genes across Clusters of Orthologous Groups (COG) functional categories for the four analyzed *Paraburkholderia sacchari* strains

To place the genomic features of *Paraburkholderia sacchari* LMG 19450ᵀ into an intraspecific context, a pangenome analysis was performed using all four publicly available *P. sacchari* genomes. This analysis aimed to distinguish conserved (core) from variable (accessory) genes and to determine whether genomic features considered during the biosafety assessment were shared across the species or restricted to particular strains. The pangenome of *P. sacchari* was reconstructed using anvi’o v8 based on four genomes, resulting in a total of 8,654 gene clusters. Of these, 4,782 clusters were shared by all genomes and constituted the core genome. The remaining clusters were classified as accessory (shell) or strain-specific (cloud), with distinct distributions observed among the four strains (Fig. 2).

Across the four genomes, the number of shell gene clusters ranged from 460 in strain ABIP2689 to 1,097 in strain PAR1, whereas the number of cloud gene clusters varied more markedly. Strain LMG 19450^T^ contained 635 cloud gene clusters, PAR1 harbored 498, Suichang626 encoded 522, and ABIP2689 displayed the largest number of strain-specific clusters, totaling 900 (Fig. 2B). Notably, ABIP2689 also exhibited the highest level of assembly fragmentation among the analyzed genomes.

To further characterize strain-specific gene content, cloud genes were functionally classified according to COG categories, and their distribution was compared among strains (Fig. 2C). While all strains exhibited unique genes spanning multiple functional categories, distinct trends were observed. In strain LMG 19450^T^, unique genes were mainly associated with carbohydrate transport and metabolism (G), transcription (K), amino acid transport and metabolism (E), and energy production and conversion (C). Strains PAR1 and Suichang626 showed similar functional profiles, with notable enrichment of unique genes related to transcription (K), replication, recombination and repair (L), amino acid transport and metabolism (E), and mobilome-associated functions (X). In contrast, strain ABIP2689 displayed a pronounced increase in unique genes across several functional categories, particularly energy production and conversion (C), carbohydrate transport and metabolism (G), lipid transport and metabolism (I), transcription (K), cell wall/membrane/envelope biogenesis (M), secondary metabolite biosynthesis, transport and catabolism (Q), and mobilome-related functions (X). All cloud genes identified in the pangenome analysis are listed in Supplementary Table S3.

### *In silico* screening of virulence-associated and antimicrobial resistance genes

**Fig. 3:**
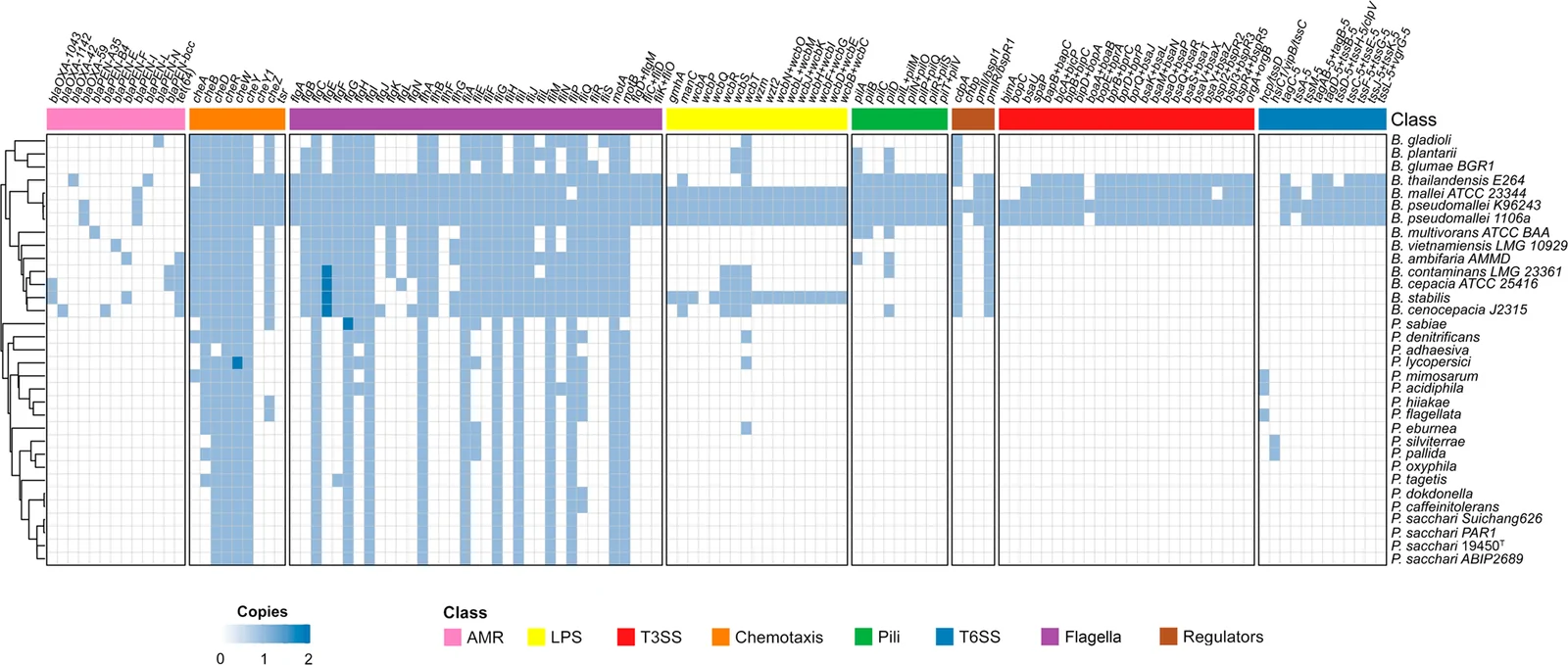
Heatmap representing the distribution and copy number of genes from *Paraburkholderia* and *Burkholderia* strains related to virulence, environmental adaptation, and host interaction across genomes included in the ANI analysis. Rows correspond to individual strains and columns represent genes, grouped by functional class and hierarchically clustered based on their presence–absence profiles. Color intensity reflects gene copy number (0–2 copies). Genes were classified into seven functional categories based on annotation and known biological roles: **antimicrobial resistance (AMR)** genes, mainly encoding efflux pumps and resistance-associated determinants; **chemotaxis** genes, involved in signal transduction and directed motility in response to environmental cues; **flagellar** genes, responsible for flagellum biosynthesis and bacterial motility; **lipopolysaccharide (LPS)** biosynthesis genes, associated with outer membrane structure and host immune interaction; **pili** genes, related to adhesion, surface attachment, and biofilm formation; **regulatory** genes, including transcriptional regulators and two-component systems controlling virulence and environmental responses; and genes encoding components of **Type III (T3SS)** and **Type VI (T6SS) secretion systems**, which mediate interactions with eukaryotic hosts or competing bacteria. Abbreviations: P., *Paraburkholderia*; B., *Burkholderia*

An extensive in silico screening for virulence-associated genes and other traits commonly linked to pathogenicity was performed across all genomes included in the ANI analysis, excluding *Ralstonia* species, in order to compare environmental *Paraburkholderia* strains with pathogenic *Burkholderia* representatives (Fig. 3). The presence or absence of genes related to secretion systems, surface structures, toxins, adhesion, intracellular survival, and stress response was assessed, and results are summarized in a binary heatmap.

Overall, pathogenic *Burkholderia* species exhibited a broad and consistent repertoire of virulence-associated genes, including components of specialized secretion systems, lipopolysaccharide biosynthesis clusters, and multiple factors associated with host interaction and immune evasion (Fig. 3). In contrast, in general genomes of the genus *Paraburkholderia* showed a markedly reduced set of such genes. When present, virulence-associated genes were primarily limited to functions related to general cellular processes, such as membrane integrity, stress tolerance, and basic secretion machinery, rather than to canonical pathogenicity determinants. The gene presence–absence patterns observed in *P. sacchari* strains were highly consistent across strains and clearly distinct from those of pathogenic *Burkholderia* species included for comparison.

Antimicrobial resistance genes were further investigated using the Resistance Gene Identifier (RGI) against the CARD database, focusing exclusively on *Burkholderia* and *Paraburkholderia* genomes. Across all *P. sacchari* strains, a limited and conserved set of five resistance-associated hits was detected under strict criteria (Supplementary data - Table S5). These included one small multidrug resistance (SMR) efflux pump homolog (*qacG*) and four resistance–nodulation–cell division (RND) efflux pump homologs corresponding to *adeF*. All identified resistance determinants were associated with antibiotic efflux mechanisms and showed moderate to high sequence similarity to reference entries, with percent identity values ranging from approximately 41.7% to 79.6% and coverage close to or exceeding full-length reference sequences.

Notably, the resistance profiles inferred by CARD were identical across the analyzed *P. sacchari* genomes and did not include genes encoding antibiotic-modifying enzymes, target-altering mutations, or high-confidence resistance determinants typically associated with multidrug-resistant pathogenic *Burkholderia*. These results indicate a conserved and limited resistome dominated by efflux-related mechanisms among *P. sacchari* strains.

To further evaluate the genomic features of *Paraburkholderia sacchari* in the context of biosafety and potential clinical concern, secondary metabolite biosynthetic gene clusters (BGCs) were identified using antiSMASH across all four available *P. sacchari* genomes. This analysis aimed to characterize the biosynthetic potential of the four genomes and identify biosynthetic gene clusters that could provide additional context for the genomic biosafety assessment.

**Table 2:** Distribution of predicted biosynthetic gene cluster (BGC) classes among four Paraburkholderia sacchari genomes

BGC class	Putative biological function	Strains (number of predicted BGCs)			
		LMG 19450ᵀ	PAR1	Suichang 626	ABIP2689
Terpene	Biosynthesis of terpenoid metabolites potentially involved in oxidative stress protection, membrane stability, and ecological interactions	4	4	4	4
Terpene precursor	Biosynthesis of isoprenoid precursors for terpene metabolism	1	1	1	1
RiPP-like	Biosynthesis of ribosomally synthesized and post-translationally modified peptides potentially involved in microbial competition and signaling	3	3	3	3
Redox cofactor	Biosynthesis of redox-active cofactors involved in specialized metabolic pathways	1	1	1	2
NRPS-like	Biosynthesis of non-ribosomal peptide-like specialized metabolites	1	1	1	2
NRPS/T1PKS hybrid	Biosynthesis of hybrid non-ribosomal peptide/polyketide specialized metabolites	1	1	1	1
HR-T2PKS	Biosynthesis of aromatic polyketides	1	1	1	1
Arylpolyene	Biosynthesis of arylpolyene pigments associated with protection against oxidative stress	1	1	1	1
Phosphonate	Biosynthesis of phosphonate-containing specialized metabolites	1	1	1	1
Hydrogen cyanide	Biosynthesis of hydrogen cyanide, a metabolite associated with microbial competition	2	1	1	0
Hserlactone	Biosynthesis of homoserine lactones involved in quorum sensing	1	1	2	1
Betalactone	Biosynthesis of β-lactone specialized metabolites	0	1	1	0

The distribution of individual biosynthetic gene clusters across the four *P. sacchari* genomes is summarized in Table 2. antiSMASH analysis predicted 18 to 19 biosynthetic gene clusters (BGCs) across the four *P. sacchari* genomes, representing 12 biosynthetic classes. Terpene-associated BGCs were the most abundant, with four predicted clusters identified in each genome. RiPP-like BGCs (three per genome), terpene precursor, NRPS/T1PKS hybrid, HR-T2PKS, arylpolyene, and phosphonate BGCs were consistently detected across all strains. Variation among genomes was limited to a small number of BGC classes, including redox cofactor and NRPS-like clusters, which were represented by two predicted BGCs in strain ABIP2689, hydrogen cyanide clusters, which were absent from ABIP2689 but present in one or two copies in the remaining strains, Hserlactone clusters, with two copies identified in strain Suichang626, and betalactone clusters, which were detected exclusively in strains PAR1 and Suichang626.

Comparison against the MiBIG database identified only two low-confidence similarities to previously characterized biosynthetic gene clusters, corresponding to the HR-T2PKS cluster (TP-1161) and the Hserlactone cluster (bacillomycin D). No significant similarities to experimentally characterized reference clusters were detected for the remaining predicted BGCs.

### Horizontal gene transfer dynamics across *Burkholderia* and *Paraburkholderia*

**Fig. 4:**
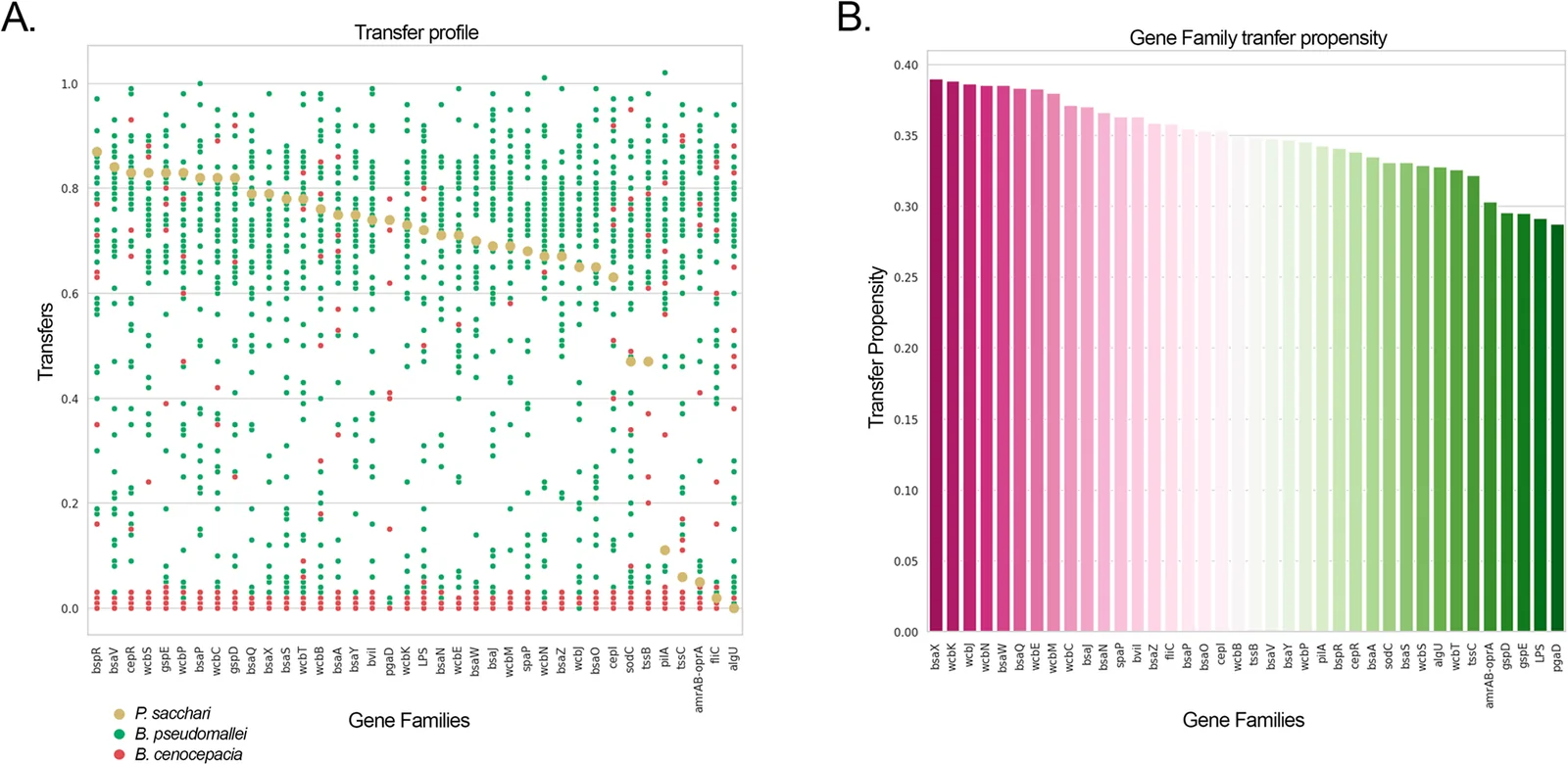
Horizontal gene transfer propensity of gene families associated with pathogenicity, host interaction, environmental persistence, and antimicrobial resistance. (**A**) Distribution of normalized horizontal gene transfer (HGT) indices inferred by gene tree-species tree reconciliation (AleRax) for 41 selected gene families across *Paraburkholderia sacchari* (yellow), *Burkholderia pseudomallei* (green), and *Burkholderia cenocepacia* (red). Each small point represents the normalized transfer index estimated for a single genome, whereas the larger yellow circles indicate the mean value for *P. sacchari*. Gene families are arranged from left to right according to the mean transfer propensity observed in *P. sacchari*. Higher values indicate gene families with stronger evidence of horizontal transfer. (**B**) Mean normalized HGT propensity for each gene family calculated across all genomes in which that family was detected. Gene families are ordered from highest to lowest transfer propensity, highlighting differences in the evolutionary mobility of the selected pathogenicity-associated genes

To complement the genomic biosafety assessment, we investigated the evolutionary history of gene families previously associated with pathogenicity, host interaction, environmental persistence, and antimicrobial resistance in *Burkholderia*. A total of 41 gene families representing these functions were selected for gene tree–species tree reconciliation analysis. For each gene family, reconciliation profiles were inferred based on phylogenetic gene trees and a reference species tree, allowing the quantification of origination, duplication, loss, and horizontal gene transfer (HGT) events across *Burkholderia* lineages (Fig. 4).

The 41 gene families selected for reconciliation analysis encompass functions commonly associated with host interaction, surface structure biogenesis, secretion systems, stress tolerance, and antimicrobial resistance. These include genes involved in lipopolysaccharide and capsular polysaccharide biosynthesis (e.g., *wcb*, *wcbE*, *wcbM*, *wcbI*), secretion system components and effectors (e.g., *bsa*, *bsaZ*, *bsaN*, *bsaQ*), adhesion and pili assembly (e.g., *pilA*, *tsdB*), transcriptional regulation (e.g., *bspR*, *wcbR*), as well as efflux and resistance-associated genes (e.g., *amrAB-oprA*). Together, these gene families represent a broad spectrum of functions previously implicated in pathogenicity, environmental persistence, and niche adaptation within the genus *Burkholderia*.

Overall, reconciliation analysis revealed contrasting horizontal gene transfer profiles between pathogenic *Burkholderia* species and *P. sacchari*. Gene families from pathogenic *Burkholderia* species, including *B. pseudomallei* and *B. cenocepacia*, displayed consistently higher inferred transfer frequencies across multiple virulence-associated gene families. In these species, several gene families exhibited elevated HGT signals, with transfer values frequently exceeding 0.7 for genes related to secretion systems, surface structures, and host interaction.

In contrast to the pathogenic *Burkholderia* species analyzed, *P. sacchari*, gene families classically associated with virulence and antimicrobial resistance, including components of the type III secretion system (*bsa*), type VI secretion system (*tss*), and the multidrug efflux operon *amrAB-oprA*, were either absent or exhibited low transfer indices. For these loci, inferred transfer frequencies were consistently close to zero or below 0.2.

Across the analyzed gene set, elevated transfer frequencies were predominantly associated with pathogenic *Burkholderia* species and genes related to secretion systems, surface polysaccharides, and membrane-associated functions, whereas *P. sacchari* consistently displayed lower transfer indices for the same functional categories.

To further characterize gene mobility independently of species identity, a gene-centered analysis was performed by calculating the mean transfer propensity for each gene family across all genomes in which it was detected (Fig. 4B). This analysis revealed that the most transfer-prone gene families were predominantly associated with capsule biosynthesis and surface polysaccharide loci, including *bsaX*, *wcbK*, *wcbJ*, and *wcbW*, as well as selected components of the type III secretion system such as *bsaN* and *bsaQ*. These gene families displayed mean transfer indices approaching 0.40, indicating frequent horizontal acquisition across the analyzed genomes.

### Phenotypic antibiotic susceptibility of *Paraburkholderia sacchari*

Antibiotic susceptibility was assessed by disk diffusion following CLSI guidelines. Susceptibility profiles were determined for *Paraburkholderia sacchari* strain LMG 19450^T^ and compared with *Pseudomonas putida* KT2440 and *Burkholderia cepacia* as reference strains (Table 3).

**Table 3:** Disk diffusion inhibition zone diameters (mean ± SD, mm) and susceptibility (S) or resistance (R) to different antibiotics

Antibiotic	*P*. sacchari LMG 19450^T^	B. cepacia B008/25,416	*P*. putida KT2440
Penicillin (PEN)	50.00 ± 0.00 (S)	0.00 ± 0.00 (R)	0.00 ± 0.00 (R)
Tetracycline (TET)	40.67 ± 3.06 (S)	0.00 ± 0.00 (R)	21.83 ± 0.55 (R)
Ceftazidime (CAZ)	22.67 ± 1.15 (S)	33.23 ± 0.72 (S)	31.57 ± 0.74
Trimethoprim–Sulfamethoxazole (SXT)	34.67 ± 2.31 (S)	30.73 ± 1.27 (S)	0.00 ± 0.00 (R)
Amoxicillin–Clavulanic Acid (AMC)	49.33 ± 1.15 (S)	0.00 ± 0.00 (R)	27.23 ± 2.16
Ciprofloxacin (CIP)	39.33 ± 1.15	0.00 ± 0.00 (R)	0.00 ± 0.00 (R)
Meropenem (MEM)	30.00 ± 0.00 (S)	32.13 ± 1.78 (S)	33.30 ± 1.47 (S)
Imipenem (IPM)	19.33 ± 1.15 (S)	0.00 ± 0.00 (R)	31.53 ± 0.60
Doxycycline (DOX)	41.33 ± 2.31	27.23 ± 1.97	23.17 ± 1.12
Chloramphenicol (CLO)	35.33	32.27 ± 0.74	0.00 ± 0.00
Nalidixic Acid (NAL)	17.33 ± 4.62	NA	NA
Levofloxacin (LVX)	32.00 ± 3.46	NA	NA

Distinct susceptibility patterns were observed among the three strains. *B. cepacia* exhibited the broadest resistance profile, showing complete resistance to penicillin (PEN), tetracycline (TET), amoxicillin–clavulanic acid (AMC), ciprofloxacin (CIP), and imipenem (IPM), while remaining susceptible to ceftazidime (CAZ), trimethoprim–sulfamethoxazole (SXT), and meropenem (MEM). *P. putida* KT2440 displayed an intermediate profile, with resistance to PEN, TET, SXT, and CIP, while remaining susceptible to MEM. In contrast, *P. sacchari* LMG 19450^T^ showed susceptibility to most antibiotics tested, including PEN, TET, CAZ, SXT, AMC, MEM, and IPM. The largest inhibition zones were observed for PEN (50.0 ± 0.0 mm), AMC (49.3 ± 1.2 mm), DOX (41.3 ± 2.3 mm), TET (40.7 ± 3.1 mm), and CIP (39.3 ± 1.2 mm).

For some antibiotics, inhibition zone diameters fell within intermediate ranges for which a clear classification as susceptible or resistant could not be established according to the adopted criteria. These cases are reported in Table 2 without susceptibility annotations and include CIP, DOX, and CLO for *P. sacchari*; CAZ, AMC, DOX, and CLO for *P. putida*; and DOX and CLO for *B. cepacia*. Nalidixic acid (NAL) and levofloxacin (LVX) were evaluated exclusively for *P. sacchari* and were therefore not assessed for the reference strains.

### Quantitative antimicrobial susceptibility profiling by broth microdilution

To further quantitatively evaluate intrinsic antimicrobial susceptibility, minimum inhibitory concentrations (MICs) were determined by broth microdilution for selected antibiotic classes (Table 4). Consistent with the disk diffusion assays, *Paraburkholderia sacchari* displayed markedly lower MIC values than *Burkholderia cepacia* across multiple antibiotic classes, including aminoglycosides, carbapenems, tetracyclines, and β-lactams.

**Table 4:** Minimum inhibitory concentrations (MIC) values determined by broth microdilution for *Paraburkholderia sacchari* LMG 19450^T^, *Burkholderia cepacia* B008/25,416, and *Pseudomonas putida* KT2440

Antibiotic	*P*. sacchari LMG 19450^T^ (µg/ml)	B. cepacia B008/25,416 (µg/ml)	*P*. putida KT2440 (µg/ml)
Piperacillin	1	4	16
Meropenem	4	64	32
Gentamicin	0,25	32	0,5
Ciprofloxacin	2	8	0,5
Doxycycline	0,25	4	0,125
Chloramphenicol	8	16	32
Fosfomycin	256	> 1024	1024

Particularly pronounced differences were observed for gentamicin and meropenem. *P. sacchari* exhibited low MIC values for gentamicin (0.25 µg/ml), whereas *B. cepacia* displayed substantially higher values (32 µg/ml). Similarly, meropenem MIC values for *P. sacchari* remained low (4 µg/ml), contrasting with the elevated ones observed for *B. cepacian* (64 µg/ml) and *Pseudomonas putida* KT2440 (32 µg/ml). In several cases, *P. sacchari* also exhibited susceptibility profiles comparable to or more favorable than those observed for *P. putida* KT2440. Lower MIC values were consistently observed for piperacillin, chloramphenicol, and doxycycline in *P. sacchari* relative to *P. putida*.

Both *B. cepacia* and *P. putida* exhibited high MIC values for fosfomycin, whereas *P. sacchari* showed comparatively lower inhibitory concentrations, although still elevated relative to the other antibiotics tested. Overall, the MIC profiles reinforce the reduced intrinsic resistance phenotype of *P. sacchari* and further distinguish this species from multidrug-resistant representatives of the *Burkholderia cepacia* complex.

### Potential infectivity of *P. sacchari* on *Galleria mellonella* infection model assays

**Fig. 5:**
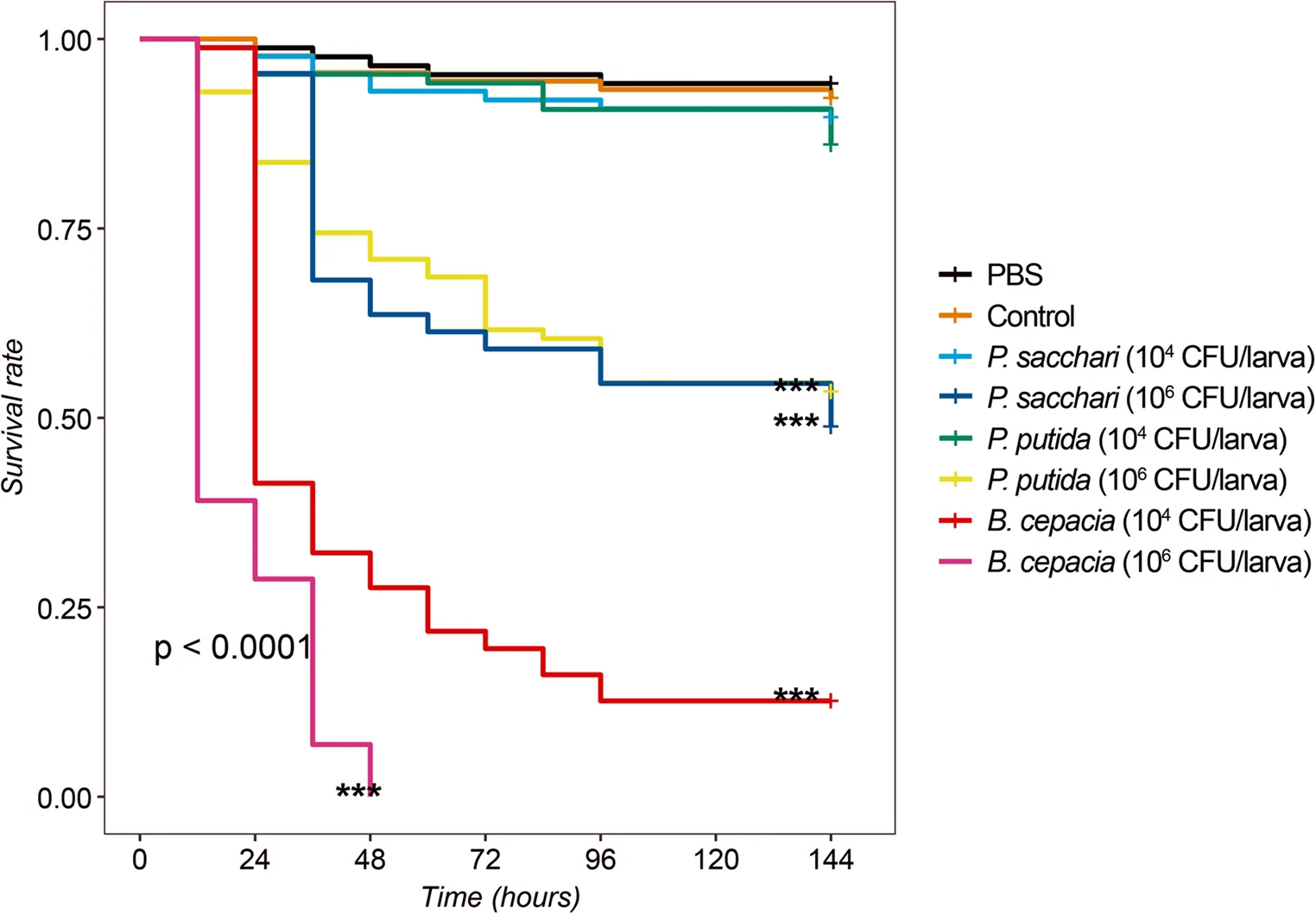
Survival of *Galleria mellonella* larvae following bacterial infection. Kaplan–Meier survival curves of *G. mellonella* larvae infected with *Paraburkholderia sacchari* strain LMG 19450^T^ (*P. sacchari*), *Pseudomonas putida* KT2440 (*P. putida*), and *Burkholderia cepacia* B008/25,416 (B. *cepacia*). Phosphate-buffered saline (PBS) and non-inoculated larvae (Control) were used as vehicle and negative controls, respectively. Larvae were inoculated with 10 µL of bacterial suspension at either 10^6^ CFU/larva or 10^4^ CFU/larva and incubated at 28 °C. Survival was monitored for 144 h. Statistical significance was assessed using the log-rank test (*p* < 0.0001)

The virulence potential of *Paraburkholderia sacchari* strain LMG 19450^T^ was evaluated using a *Galleria mellonella* infection model and compared with *Pseudomonas putida* KT2440 and *Burkholderia cepacia* B008/25,416 as reference strains (Fig. 5).

Larvae inoculated with *B. cepacia* showed rapid mortality, with survival decreasing sharply during the first 48 h post-infection and no surviving larvae remaining by the end of the second day. In contrast, larvae infected with *P. sacchari* strain LMG 19450^T^ exhibited substantially higher survival rates throughout the experiment.1.

The survival profile of larvae infected with *P. sacchari* LMG 19450^T^ was comparable to that observed for *P. putida* KT2440. In both cases, a fraction of larvae remained alive until the end of the monitoring period, indicating limited virulence under the experimental conditions tested. Statistical analysis of survival curves revealed significant differences between the *B. cepacia* group and the other treatments (log-rank test, *p* < 0.0001).

Control groups inoculated with phosphate-buffered saline (vehicle control) or left non-inoculated showed no mortality during the experiment, confirming the suitability of the infection model and experimental conditions.

## Discussion

Members of the family Burkholderiaceae exemplify how bacterial lineages can diverge profoundly in ecological function and biological impact while remaining closely related at the phylogenetic level. Since its original description, the genus *Burkholderia* has been associated simultaneously with severe human disease, particularly in immunocompromised hosts, and with remarkable metabolic versatility in soil and plant-associated environments. This coexistence of pathogenic and environmental lifestyles within a single taxonomic framework has long complicated both evolutionary interpretation and practical decision-making regarding biosafety and application. Genome-scale phylogenetic analyses over the past decade have progressively clarified this complexity, revealing that pathogenic and environmental species form deeply separated evolutionary lineages, ultimately motivating the establishment of the genus *Paraburkholderia* to encompass predominantly non-pathogenic, plant-associated taxa (Sawana et al. 2014; Dobritsa and Samadpour 2016). Within this context, *Paraburkholderia sacchari* represents a paradigmatic environmental species whose biology is tightly linked to plant-associated soils and whose biotechnological potential has been recognized for more than two decades.

The present study places *P. sacchari* strain LMG 19450^T^ within this evolutionary and ecological framework using a combination of high-resolution genomics and functional assays. The generation of a near-complete genome assembly was a critical prerequisite for this effort. Burkholderiaceae genomes are among the most challenging bacterial genomes to assemble, owing to their high GC content, frequent repetitive sequences, expansive gene families, and complex multipartite organization. Fragmented assemblies can obscure or artificially inflate gene counts, disrupt operon structures, and confound comparative analyses of virulence and resistance loci (Klassen and Currie et al. 2012; diCenzo and Finan 2017; Vandamme et al. 2017). By resolving the genome of strain LMG 19450^T^ to high contiguity and completeness, we were able to confidently assess gene content, structural features, and evolutionary relationships, thereby avoiding many of the pitfalls that have historically complicated genomic studies of this group.

Phylogenetic reconstruction based on 16 S rRNA sequences, combined with genome-wide similarity metrics, unambiguously places strain LMG 19450^T^ within the *Paraburkholderia* lineage and separates it from pathogenic *Burkholderia* species. This placement is consistent with previous phylogenomic analyses demonstrating that environmental *Paraburkholderia* species form a coherent clade distinct from *Burkholderia sensu stricto*, which contains most clinically relevant taxa (Sawana et al. 2014; Estrada-de los Santos et al. 2018). While ANI values strongly support the classification of all four analyzed strains as *P. sacchari*, the observed variation in dDDH values, particularly for strain ABIP2689, highlights the genomic heterogeneity that can exist within environmental bacterial species. Such heterogeneity has been repeatedly documented in soil-associated Burkholderiaceae and likely reflects a combination of ecological specialization, geographic separation, and differential exposure to horizontal gene transfer, rather than discrete species boundaries (Rosselló-Móra and Amann 2015; Chun et al. 2018). Therefore, ANI and dDDH analyses primarily served to establish the taxonomic framework required for meaningful genomic comparisons with closely related environmental *Paraburkholderia* strains.

This genomic diversity is further reflected in the pangenome architecture of *P. sacchari.* The presence of a large, conserved core genome, together with an extensive accessory and strain-specific gene pool, is consistent with an open pangenome, a feature commonly observed in bacteria occupying heterogeneous and dynamic environments such as plant-associated soils. Similar patterns have been reported for numerous *Paraburkholderia* species, in which accessory genes contribute to metabolic flexibility, stress tolerance, and niche adaptation rather than to host invasion or virulence (Tettelin et al. 2008; Bach et al. 2021). Notably, all currently available *P. sacchari* strains were isolated from plant-associated soils, including sugarcane, tomato, bamboo roots, and rice fields. This consistent association with the rhizosphere may suggests that the genomic variability observed among strains reflects adaptation to specific plant hosts and soil chemistries, a hypothesis supported by studies showing that *Paraburkholderia* species frequently function as plant growth-promoting bacteria and, in some cases, as biological control agents in agricultural systems (Estrada-de los Santos et al. 2018; Rojas-Rojas et al. 2025).

The functional composition of the strain-specific gene pool reinforces this ecological interpretation. Unique genes were predominantly associated with metabolic processes, membrane and cell envelope biogenesis, regulatory functions, and transport systems, categories commonly implicated in nutrient acquisition, environmental sensing, and interaction with plant-derived compounds. The enrichment of genes involved in transport and metabolic functions is particularly consistent with adaptation to plant-associated environments, where availability of carbon sources, root exudates, and microbial competitors can vary substantially among host species (Jacoby and Kopriva 2018; Mavrodi et al. 2021). Similar functional enrichments have been described in pangenomic studies of other plant-associated *Paraburkholderia* and differ from patterns commonly reported for pathogenic *Burkholderia*, where strain-specific regions are often enriched in virulence determinants, secretion systems and host interaction factors (Holden et al. 2008; Bodilis et al. 2018; Jia and Lu 2024). Thus, the pangenome of *P. sacchari* appears to be shaped primarily by ecological pressures imposed by plant-associated lifestyles rather than by selective forces favoring pathogenicity. Rather than constituting direct evidence of biosafety, this analysis provided the evolutionary context necessary to evaluate whether genomic features considered during the biosafety assessment, including genes associated with virulence or host interaction, were conserved within the species or restricted to particular lineages.

This ecological specialization provides an important lens through which to interpret the extensive virulence gene screening performed in this study. The large presence/absence matrix derived from VFDB and NCBI searches reveals a clear dichotomy between pathogenic *Burkholderia* species and *P. sacchari*. While pathogenic strains harbor dense repertoires of secretion systems, surface structures, and host-interaction genes, *P. sacchari* exhibits a markedly reduced and conserved set of such loci. Recent comparative genomic studies have shown that the distinction between environmental and pathogenic Burkholderiaceae is often not defined by the presence of a few individual virulence factors, but rather by broader genomic signatures associated with different ecological lifestyles (Guerrero-Egido et al. 2024). In this context, the consistent reduction of virulence-associated loci across all analyzed P. sacchari strains supports the view that the species belongs to a lineage whose evolutionary trajectory has been shaped primarily by adaptation to plant-associated environments.

Because horizontal gene transfer is a major mechanism driving the acquisition and dissemination of virulence-associated traits in Burkholderia, we investigated the evolutionary histories of gene families associated with pathogenicity, host interaction, environmental persistence, and antimicrobial resistance. This analysis provides an evolutionary framework for interpreting whether these functions have been predominantly maintained through vertical inheritance or frequently disseminated by horizontal transfer. Pathogenic *Burkholderia* species are well known for their genomic plasticity and consistent with this model, the gene reconciliation analyses performed here revealed elevated transfer frequencies among virulence-associated gene families in pathogenic species. Similar observations have been reported for several pathogenic Burkholderia species, in which genomic islands and other horizontally acquired regions contribute substantially to the distribution of secretion systems, regulatory networks, and host-interaction determinants associated with pathogenicity (Khrongsee et al. 2024; Bodilis et al. 2018; Tuanyok et al. 2008). Consistent with these observations, capsule biosynthesis and surface polysaccharide loci displayed the highest transfer propensities among the gene families analyzed. Previous studies have shown that these loci are frequently located within genomic islands or associated with mobile genetic elements, facilitating their horizontal dissemination and structural diversification (Losada et al. 2010). Together, these observations support the view that horizontal gene transfer has been an important mechanism driving the assembly and diversification of virulence-associated repertoires within pathogenic Burkholderia lineages. In contrast, *P. sacchari* displayed substantially more constrained transfer dynamics. This pattern is consistent with the reduced and highly conserved virulence gene repertoire observed across all analyzed strains, suggesting that pathogenicity-associated functions are not undergoing the same degree of horizontal expansion observed in pathogenic relatives. Similar trends have been reported for environmental and plant-associated Burkholderiaceae, in which genomic diversification appears to be more strongly associated with ecological adaptation and metabolic specialization than with recurrent acquisition of host-associated traits (Bottery 2022). The comparatively limited contribution of horizontal transfer to virulence-associated gene families in *P. sacchari* therefore provides an evolutionary explanation for the stability of its low-virulence genomic profile despite the broader genomic diversity observed within the species.

The identification of multiple secondary metabolite biosynthetic gene clusters in *P. sacchari* is consistent with the repertoire commonly reported for environmental members of the genera *Paraburkholderia*. The predominance of terpene, RiPP-like, phosphonate, and arylpolyene clusters is consistent with the metabolic profiles commonly observed in environmental *Burkholderia* and *Paraburkholderia* species, where such compounds are frequently linked to microbial competition, stress tolerance, signaling, and interactions within the rhizosphere (Kim et al. 2024; Klaus et al. 2020). Comparison against the MiBIG database identified only two low-confidence similarities to previously characterized BGCs, while no high-confidence matches to experimentally characterized toxin biosynthetic pathways were detected. Although bioinformatic predictions alone cannot establish metabolite production or biological function, the predicted BGC repertoire is consistent with the low pathogenic potential indicated by the comparative genomic analyses, antimicrobial susceptibility profile, and *Galleria mellonella* infection assays. Together, these observations support the view that the predicted secondary metabolite repertoire of *P. sacchari* resembles that of other environmental *Paraburkholderia* species and does not reveal genomic features typically associated with specialized toxin biosynthesis. Instead, the detected biosynthetic potential aligns with traits desirable in biotechnological and agricultural applications, such as the production of antimicrobial or signaling compounds that may contribute to plant protection, bioinsecticidal activity, or competitive fitness in soil environments (Song et al. 2024).

Antimicrobial resistance further accentuates the contrast between pathogenic and environmental lineages. Members of the *Burkholderia cepacia* complex are notorious for intrinsic and acquired multidrug resistance, a trait that contributes substantially to their clinical significance (Mahenthiralingam and Vandamme 2005). In contrast, *P. sacchari* harbors only a limited set of efflux-based resistance determinants and exhibits broad phenotypic susceptibility under standardized testing conditions. This genotype–phenotype concordance is consistent with observations in other environmental *Burkholderia* and *Paraburkholderia* isolates and reflects the absence of sustained antibiotic selective pressure in plant-associated soils (Nesme and Simonet 2014; Angus et al. 2014; Stanton et al. 2020). Quantitative MIC profiling further reinforced this distinction. Across multiple antibiotic classes, including aminoglycosides, carbapenems, tetracyclines, and β-lactams, *P. sacchari* consistently displayed substantially lower inhibitory concentrations than *B. cepacia*. Particularly strong differences were observed for gentamicin and meropenem, antibiotics commonly associated with intrinsic resistance mechanisms in *Burkholderia cepacia* complex strains. Importantly, the susceptibility profile of *P. sacchari* was not only markedly distinct from *B. cepacia*, but in several cases also comparable to or more favorable than that of *Pseudomonas putida* KT2440, a benchmark microbial chassis widely regarded as safe and genetically tractable for industrial biotechnology applications (Kampers et al. 2019). These findings strengthen the interpretation that *P. sacchari* lacks the characteristic multidrug-resistant phenotype commonly associated with pathogenic *Burkholderia* lineages. Instead, its antimicrobial susceptibility profile is more consistent with environmentally adapted bacteria subjected to limited antibiotic selective pressure, reinforcing its suitability as a lower-risk non-conventional microbial chassis.

The biological relevance of these genomic patterns is underscored by the *Galleria mellonella* infection assays. The stark difference in larval mortality between *B. cepacia* and *P. sacchari* mirrors the genomic divergence observed across virulence, resistance, and horizontal gene transfer analyses. Importantly, the virulence profile of *P. sacchari* closely resembled that of *P. putida* KT2440. The similarity between *P. sacchari* LMG 19450^T^ and *P. putida* KT2440 in antibiotic susceptibility and attenuated virulence provides an important point of reference, indicating that *P. sacchari* shares phenotypic characteristics with an environmental bacterium that has been extensively used as a microbial chassis in biotechnology (Lorenzo et al. 2024; Martínez-García and de Lorenzo 2024; Belda et al. 2016).

Taken together, these findings converge on a coherent interpretation: *P. sacchari* is not an anomalous or borderline member of a pathogenic genus, but rather a representative of a distinct environmental lineage whose evolution has been shaped by persistent association with plant-derived niches. Its genome reflects adaptation to soil and rhizosphere environments through metabolic flexibility and regulatory diversity, rather than through the acquisition of virulence traits. Phylogenomic analyses further support the placement of this species within the environmental *Paraburkholderia* lineage, consistent with its current taxonomic designation as *Paraburkholderia sacchari*. By integrating high-quality genomics with comparative, evolutionary, and phenotypic analyses, this study provides a comprehensive and internally consistent framework for evaluating the biosafety and biotechnological potential of *P. sacchari*. The concordance among phylogenomic, comparative genomic, antimicrobial susceptibility, and *G. mellonella* infection assay data consistently supports the environmental lifestyle and low pathogenic potential of the species. Nevertheless, despite its widespread use as an in vivo model for evaluating bacterial virulence, the immune system of *G. mellonella* differs substantially from that of vertebrates, particularly due to the absence of adaptive immunity, limiting the direct extrapolation of infection outcomes to mammalian hosts (Tsai et al. 2016; Ménard et al. 2021). Accordingly, the infection assays presented here should be viewed as one component of the overall biosafety assessment, providing evidence consistent with the low pathogenic potential of *P. sacchari* when interpreted alongside the comparative genomic and antimicrobial susceptibility analyses. Future studies incorporating broader strain collections, functional characterization of accessory genes, and complementary vertebrate infection models would further strengthen the evidence supporting the biosafety profile of *P. sacchari* and expand our understanding of its ecological diversification and potential for safe biotechnological applications.

## Conclusions

This study provides a comprehensive genomic and phenotypic assessment of *Paraburkholderia sacchari* strain LMG 19450^T^, situating it unambiguously within the environmental lineage of the Burkholderiaceae and clearly distinct from pathogenic *Burkholderia* species. Through high-quality genome assembly, comparative genomics, pangenome reconstruction, and phylogenetic analyses, we demonstrate that *P. sacchari* exhibits a genomic architecture shaped primarily by adaptation to plant-associated soil environments rather than by virulence-driven evolution. The absence of canonical pathogenicity determinants, the restrained horizontal gene transfer profile, and the limited resistome collectively differentiate *P. sacchari* from clinically relevant relatives and align it with other environmentally adapted *Paraburkholderia* species. Importantly, genomic predictions were corroborated by phenotypic evidence, including broad antibiotic susceptibility and attenuated virulence in the *Galleria mellonella* infection model. Under the conditions tested, *P. sacchari* strain LMG 19450^T^ displayed susceptibility profiles that were comparable to, and in some cases more favorable than, those observed for *Pseudomonas putida* KT2440, a benchmark microbial chassis widely regarded for its biosafety and robustness. Together, these findings support the characterization of *P. sacchari* strain LMG 19450^T^ as an environmentally adapted bacterium with a low pathogenic potential and strong potential for biotechnological and agricultural applications. More broadly, this work highlights the value of integrating genome-scale analyses with functional validation to inform biosafety assessment and strain selection, providing a robust framework for the responsible development of environmental bacteria as microbial chassis. These results, collectively, support the taxonomic placement of this organism within the genus *Paraburkholderia*, reinforcing the reclassification of *Burkholderia sacchari* as *Paraburkholderia sacchari* and further supporting its suitability for biotechnological development based on the genomic and phenotypic evidence presented here.

## Supplementary Information

Below is the link to the electronic supplementary material.

Supplementary Material 1

Supplementary Material 2

Supplementary Material 3

Supplementary Material 4

Supplementary Material 5

Supplementary Material 6

Supplementary Material 7

## Data Availability

The datasets generated during and/or analyzed during the current study are available from the corresponding author on request.
